# Senescence of endothelial cells promotes phenotypic changes in adventitial fibroblasts: possible implications for vascular aging

**DOI:** 10.1007/s11010-024-05028-7

**Published:** 2024-05-14

**Authors:** Katarzyna Sarad, Urszula Jankowska, Bozena Skupien-Rabian, Anne Babler, Rafael Kramann, Józef Dulak, Agnieszka Jaźwa-Kusior

**Affiliations:** 1 Department of Medical Biotechnology, Faculty of Biochemistry, Biophysics and Biotechnology, Jagiellonian University, Gronostajowa Str. 7, 30-387 Krakow, Poland; 2 Doctoral School of Exact and Natural Sciences, Jagiellonian University, Kraków, Poland; 3 Proteomics and Mass Spectrometry Core Facility, Malopolska Centre of Biotechnology, Kraków, Poland; 4 Department for Renal and Hypertensive Diseases, Rheumatological and Immunological Diseases, RWTH Aachen University, Aachen, Germany; 5 Department of Internal Medicine, Nephrology and Transplantation, Erasmus Medical Center, Rotterdam, The Netherlands

**Keywords:** Adventitial fibroblasts, Endothelial cell senescence, Ferroptosis, GDF-15, Oxidative stress

## Abstract

Aging is the most important risk factor for the development of cardiovascular diseases. Senescent cells release plethora of factors commonly known as the senescence-associated secretory phenotype, which can modulate the normal function of the vascular wall. It is currently not well understood if and how endothelial cell senescence can affect adventitial niche. The aim of this study was to characterize oxidative stress-induced endothelial cells senescence and identify their paracrine effects on the primary cell type of the adventitia, the fibroblasts. Human aortic endothelial cells (HAEC) were treated with hydrogen peroxide to induce premature senescence. Mass spectrometry analysis identified several proteomic changes in senescent HAEC with top upregulated secretory protein growth differentiation factor 15 (GDF-15). Treatment of the human adventitial fibroblast cell line (hAdv cells) with conditioned medium (CM) from senescent HAEC resulted in alterations in the proteome of hAdv cells identified in mass spectrometry analysis. Majority of differentially expressed proteins in hAdv cells treated with CM from senescent HAEC were involved in the uptake and metabolism of lipoproteins, mitophagy and ferroptosis. We next analyzed if some of these changes and pathways might be regulated by GDF-15. We found that recombinant GDF-15 affected some ferroptosis-related factors (e.g. ferritin) and decreased oxidative stress in the analyzed adventitial fibroblast cell line, but it had no effect on erastin-induced cell death. Contrary, silencing of GDF-15 in hAdv cells was protective against this ferroptotic stimuli. Our findings can be of importance for potential therapeutic strategies targeting cell senescence or ferroptosis to alleviate vascular diseases.

**Supplementary Information:**

The online version contains supplementary material available at 10.1007/s11010-024-05028-7.

## Introduction

The cells in the healthy arterial wall are arranged in three layers: intima, media, and adventitia. Unlike intima (composed of endothelial cells) and media (composed of smooth muscle cells), the adventitia contains different cell types such as endothelial cells of *vasa vasorum*, immune cells, various mesenchymal cells, as well as different vascular progenitor cells [1]. In response to injury and environmental stress, fibroblasts, the most abundant adventitial cells, start to proliferate, differentiate into myofibroblasts, and migrate to the intima [2]. Their invasion and migration into the intima, as well as increased production and secretion of extracellular matrix (ECM) proteins, contribute to extensive remodeling, intimal hyperplasia, and arterial stenosis [2]. In addition, different cytokines, chemokines and growth factors released by activated adventitial fibroblasts directly affect the phenotype of other cells residing in the vessel wall such as vascular smooth muscle cells and endothelial cells. This may additionally lead to pathological processes in the vessel wall, such as neointima formation, *vasa vasorum* expansion, immune cells recruitment and atherosclerotic plaque growth [3]. Indeed, advanced atherosclerotic plaques are characterized by a fibrous cap rich in smooth muscle cells and fibroblasts, which facilitate the buildup of the plaque via secretion of proinflammatory cytokines and an ECM [4].

Aging is one of the well-recognized risk factors for the development of atherosclerosis, a chronic inflammatory disease of large and medium-sized arteries, involving multiple cell types [5]. During aging and chronic injury, cells in the artery wall are exposed to various stressors and undergo senescence [6]. Cellular senescence is characterized by an indefinite cell cycle arrest in response to endogenous and exogenous factors, such as DNA damage, telomere shortening, oncogene activation, and oxidative stress. The implementation of senescence is associated with dynamic changes in the expression of genes encoding proteins involved in different signaling pathways leading to activation of p53 and a rise in the production of cell cycle inhibitors, such as p21 or p16, upregulation of the BCL-2 family of antiapoptotic proteins, which induce resistance to apoptosis and metabolic changes [7]. Unlike quiescent cells, which display a reversible cell cycle arrest and a low metabolic status, senescent cells are characterized by irreversible growth arrest and high metabolic activity. Unfortunately, senescent cells lack reliable and universal measurable traits and as such are defined through the combined detection of multiple markers. Biological markers used to detect cell senescence include senescence-associated β galactosidase (SA-β-gal) activity, increase of reactive oxygen species (ROS), high expression of cell cycle inhibitors, as well as activation of DNA damage responses [8]. In the case of senescent endothelial cells, additionally reduced level of endothelial nitric oxide synthase (eNOS) and nitric oxide (NO) is observed. NO deficiency and/or development of oxidative stress lead to impaired endothelial function in terms of control over vasodilation, oxidative stress, immune cell infiltration and inflammation, blood coagulation, and glucose and lipid metabolism [9].

Senescent cells are present in human atherosclerotic plaques [10] and cell senescence has been shown to promote atherosclerotic plaque formation, to accelerate established lesions, and to cause changes in plaque composition, leading to increased necrotic cores and smaller fibrous caps – signs of plaque instability [5]. This can be related to the fact that senescent cells modulate their environment by secreting inflammatory cytokines, chemokines, matrix metalloproteinases and growth factors, collectively known as the senescence-associated secretory phenotype (SASP) [11, 12]. Thus, pathological accumulation of senescent cells and SASP components may disturb proper tissue functioning leading to age-related diseases [7, 13]. However, the effects of SASP, which can affect other cells via paracrine or endocrine mechanisms [14], can be either beneficial or detrimental depending most probably on SASP composition and the local tissue microenvironment [15].

In this study, we used oxidative stress to induce premature senescence in blood vessel-lining aortic endothelial cells, as in the vascular system they are constantly exposed to high blood oxygen levels and thus to an oxidative stress-prone environment. We found that endothelial cell-derived SASP affects some elements of the metabolic and death pathways in non-senescent adventitial fibroblasts.

## Materials and methods

### Generation of human adventitial fibroblast cell line

Adventitia was isolated from human renal artery tissue that was collected from the Urology Department of the Hospital Eschweiler from patients undergoing nephrectomy due to kidney cancer. All patients provided informed consent and the study was performed in accordance with the Declaration of Helsinki. Adventitia was digested for 4 h until single cell suspension using a sterile mixture of collagenase (3 mg/ml; Sigma) and elastase (1 mg/ml; Sigma) in serum free DMEM containing 1% Penicillin–Streptomycin-Glutamine (Gibco). The isolated cells were cultured in Mesenpan Special Medium (PanBiotech) and immortalized using retroviral vectors carrying human telomerase reverse transcriptase (*hTERT*) and simian virus 40 large T antigen (*SV40LT*). Retroviral particles were produced by transient transfection of HEK293T cells using TransIT-LT1 (Mirus). Two types of amphotropic particles were generated by co-transfection of plasmids pBABE-puro-SV40-LT (Addgene #13970) or xlox-dNGFR-TERT (Addgene #69805) in combination with a packaging plasmid pUMVC (Addgene #8449) and a pseudotyping plasmid pMD2.G (Addgene #12259). Retroviral particles were 100 × concentrated using Retro-X concentrator (Clontech) 48 h post-transfection and added to the target cells for 48 h. For selection, cells were treated with puromycin (2 μg/ml) for 7 days.

### Cell culture and treatment

Primary human aortic endothelial cells (HAEC, obtained from PromoCell at passage 2) were cultured in endothelial cell growth medium (EGM-2, Lonza) composed of EBM-2 Basal Medium and EGM-2 SingleQuots Supplements and used for experiments until passages 5–10. Immortalized human adventitial fibroblasts (hAdv) were grown in DMEM HG (Biowest) supplemented with 10% fetal bovine serum (FBS, Biowest), and penicillin (100 U/ml) and streptomycin (100 µg/ml; Gibco). The hAdv cells exhibited higher α-smooth muscle actin (α-SMA) expression upon transforming growth factor β treatment confirming their potential to differentiate towards myofibroblasts (not shown). Both cell lines were cultured at 37 °C in a humidified incubator in 5% CO_2_ atmosphere. Both cell lines were passaged upon reaching approx. 90–100% confluency using 0.25% trypsin/EDTA solution (ThermoFisher Scientific). To induce cell senescence HAEC were seeded at density of either 5 × 10^4^ per each well of 24-well plate, or 1 × 10^5^ per each well of 12-well plate, or 3 × 10^5^ per each well of 6-well plate and treated with hydrogen peroxide (H_2_O_2_, Sigma-Aldrich) at a concentration range 75–300 µM. After 24 h, the medium was replaced with the fresh EGM-2 and the cells were cultured for another 48 h. Conditioned medium (CM) was collected from control and senescent HAEC growing on 6-well plate. For the proteomic analysis hAdv cells were treated for 48 h with 1 volume of CM (either control or collected from senescent HAEC) mixed with 1 volume of regular culture medium of hAdv cells. In addition, hAdv cells were stimulated for 48 h with recombinant human GDF-15 (rhGDF-15; R&D Systems, #957-GD) at concentration of 200 ng/ml. In some experiments, hAdv cells were stimulated for 48 h with rhGDF-15 and then erastin (MedChem Express) was added for the next 24 h.

### Senescence-associated β-galactosidase (SA-β-gal) staining

HAEC cells were plated in 24-well plate and treated with different concentrations of H_2_O_2_ (300 µM, 150 µM, and 75 µM). After 24 h, the medium was replaced with the fresh EGM-2 for another 48 h. After this time, control and H_2_O_2_-treated HAEC were fixed with 4% paraformaldehyde for 5 min, washed twice with PBS and incubated overnight at 37 °C with SA-β-gal staining solution (1 mg/ml X-gal, 150 mM NaCl, 2 mM MgCl_2_, 5 mM Potassium Ferricyanide, 5 mM Potassium Ferrocyanide in 400 mM citrate buffer, pH 6). The cells were washed with PBS, stained with DAPI (Sigma-Aldrich), visualized and counted using bright field microscopy (SA-β-gal-positive cells) and fluorescence microscopy (total number of cells based on DAPI-stained nuclei).

### Trypan blue staining

Quantification of viable and dead HAEC cells in suspension was assessed with Trypan Blue (0.4% in culture media) staining and manual counting with a hemacytometer.

### Quantitative RT-PCR (qPCR)

HAEC cells were plated in 24-well plate and treated with 75 µM concentration of H_2_O_2_. After 24 h, the medium was replaced with the fresh EGM-2 for another 48 h and then RNA was isolated using Total RNA Mini kit (A&A Biotechnology). cDNA was prepared using High Capacity cDNA Reverse Transcription Kit (Applied Biosystems). Quantitative PCR was performed using specific primers described in Table 1 and *AceQ* qPCR SYBR Green Master Mix (Vazyme) in StepOne Plus Real-Time PCR system (Applied Biosystems). All procedures were performed according to manufacturers’ instructions. TATA-box binding protein (*TBP*) and elongation factor 2 (*EEF2*) served as a housekeeping genes in HAEC and hAdv cells, respectively. The relative fold gene expression was calculated using the 2^−dCt^ formula.

**Table 1:** Primers used for quantitative PCR

Target	Primer sequence (5′– > 3′)
*TBP*	F: TATAATCCCAAGCGGTTTGC
	R: GCTGGAAAACCCAACTTCTG
*EEF2*	F: TCAGCACACTGGCATAGAGG
	R: GACATCACCAAGGGTGTGCA
*p21*	F: GCAGACCAGCATGACAGATT
	R: GGATTAGGGCTTCCTCTTGG
*p16*	F: CCGAATAGTTACGGTCGGAGG
	R: CACCAGCGTGTCCAGGAAG
*eNOS*	F: TTGGTGTTTGGCTGCCGATGC
	R: GGTGAACCTCCGCGGCTAGC
*Ki-67*	F: ACGCCTGGTTACTATCAAAAG
	R: CAGACCCATTTACTTGTGTTG
*GPX4*	F: ATTGGTCGGCTGGACGAG
	R: ACTTCGGTCTTGCCTCACTG
*GSS*	F: GAAAGGCGAACTAGTGTTGG
	R: AAGTCCATCTGCACAGCATA
*GSR*	F: TGAAGTTCTCCCAGGTCAAG
	R: CAACATCTGGAATCATGGTCA
*CHAC2*	F: ATGTGGGTTTTTGGTTACGG
	R: TTGTTGTGGGATCTTTTGGA
*SLC7A11*	F: TGCAGGGCGTATTATGAGGAG
	R: GCTTTGTCTTATGCTGAATTGG
*GDF-15*	F: GCTCTCAGATGCTCCTGGTG
	R: GAGTGCAACTCTGAGGGTCC

### ELISA

Human IL-6, human IL-8 and human GDF-15 levels were assessed using DuoSet ELISA (R&D Systems) in the culture media collected from control and H_2_O_2_-treated HAEC 48 h after media replacement (72 h from addition of 75 µM H_2_O_2_) according to manufacturer’s instructions. Human ferritin level was measured in hAdv cell lysates using Ferritin ELISA (DRG Diagnostics) according to manufacturer’s protocol.

### Mass spectrometry analysis

*Sample preparation* HAEC and hAdv cells were plated in 6-well plates. HAEC cells were treated with 75 µM H_2_O_2_ for 24 h and then the medium was replaced with the fresh EGM-2 for another 48 h. hAdv cells were treated with CM from control or senescent HAEC for 48 h. The cell lysates were collected in four biological replicates for each treatment (control HAEC, H_2_O_2_-treated HAEC, control CM-treated hAdv cells and hAdv cells treated with CM from senescent HAEC). Cells were washed twice with PBS and then lysed in 20 µl of lysis buffer (1% SDS, 0.1 M Tris, pH 7.5). Collected lysates were heated for 5 min at 98 °C and sonicated in the Bioruptor Pico (Diagenode; 7 cycles of 30 s/30 s ON/OFF). After this step, samples were centrifuged, and supernatants were collected. Protein samples (50 µg) were prepared for LC–MS/MS analysis using filter-aided sample preparation (FASP) protocol [16].

*Liquid chromatography and tandem mass spectrometry *(*LC–MS/MS*)*:* The peptides obtained after FASP were analyzed with a Q Exactive mass spectrometer (Thermo Fisher Scientific) coupled with a nanoHPLC (UltiMate 3000 RSLCnano System, Thermo Fisher Scientific). Peptides were loaded onto a trap column (AcclaimPep-Map100 C18, Thermo Fisher Scientific; ID 75 µm, length 20 mm, particle size 3 µm, pore size 100 Å) and then separated on a 50 cm analytical column (AcclaimPepMapRLSC C18, Thermo Fisher Scientific; ID 75 μm, particle size 2 μm, pore size 100 Å) in a 4-h gradient of acetonitrile (2–40%) in the presence of 0.05% formic acid at a flow rate of 250 nl/min. The eluting peptides were ionized in a Digital PicoView 550 nanospray source (New Objective) and analyzed by mass spectrometer in a data-dependent mode using Top12 method. The MS and MS/MS spectra were acquired with a resolution of 70,000 and 17,500, respectively. The QCloud quality control system was used for monitoring the performance of the LC–MS/MS instrumentation during the measurements [17, 18].

*Data processing*: The acquired LC–MS/MS data were processed using MaxQuant software (version 1.6.7.0) [19] and searched by an integrated Andromeda search engine [20] against the SwissProt database restricted to Homo sapiens taxonomy (20,394 sequences; downloaded on February 8, 2021). The false discovery rate (FDR) for the peptide and protein identification was set to 1%. Match between runs algorithm was enabled. Label free quantification (LFQ) was carried out. The MaxQuant output table was further processed with the use of Perseus platform (version 1.6.15.0) [21]. The protein groups identified in the decoy database, contaminants and proteins only identified by site were filtered out. The LFQ intensities were log2-transformed. Data matrix was filtered for proteins with at least 3 valid values in each group and then missing values were replaced by random numbers drawn from a normal distribution. The Student’s t-test with the permutation-based FDR set to 1% was used to reveal changes between HAEC cells treated with H_2_O_2_ and control. To detect differences between control CM-treated hAdv cells and hAdv cells treated with CM from senescent HAEC, the Student’s t-test without correction for multiple comparisons was applied (p-value < 0.05). The sets of differential proteins obtained for both comparisons were further filtered for proteins identified based on at least 2 peptides and with the abundance fold change of at least 1.2.

*Bioinformatic analysis:* The differentially expressed proteins were further classified into the KEGG molecular pathway using DAVID Bioinformatics Resources (https://david.ncifcrf.gov/home.jsp; version 2021) [22] to identify specific biological pathways affected by cellular senescence. The highest statistical significance of protein enrichment within the respective category for HAEC (based on FDR) and for hAdv cells (based on p-value) are presented as plots drawn using the “ggplot2” packages in the R software (version 4.2.1). In addition, predicted protein–protein interactions for the list of differentially expressed proteins and the resulting network were retrieved and constructed using the STRING database version 9.0 (http://string-db.org).

### Cell viability

HAEC cells and hAdv cells were plated in 96-well plates at density of 1 × 10^4^ per each well. HAEC cells were treated with different concentrations of H_2_O_2_ (300 µM, 150 µM and 75 µM), whereas hAdv cells were treated with 200 ng/ml of GDF-15 or 0.5–5 µM of erastin. Cell viability was determined with MTT assay after 48 h. The cells were incubated with Thiazolyl Blue Tetrazolium Bromide (MTT, Sigma-Aldrich) in culture media at a concentration of 0.5 mg/ml for 4 h. The formazan crystals were dissolved with acidified isopropanol and the absorbance was measured at 562 nm using a Tecan Infinite M200 microplate reader.

### Cytotoxicity assay

Cell damage (LDH release to culture medium) was measured using CytoTox 96™ Nonradioactive Cytotoxicity Assay (Promega) according to the manufacturer’s specifications.

### Flow cytometry analysis

The hAdv cells were grown in 12-well plates, stimulated with GDF-15 or/and erastin as described above.

*Reactive oxygen species *(*ROS*)* detection* The estimation of reactive oxygen species was performed with CellRox Deep Red Reagent (Invitrogen) according to the manufacturer’s procedure. The cells were stained with 4’,6-diamidino-2-phenylindoledye (DAPI) to exclude the DAPI-positive (DAPI +) dead cells.

*Iron*(*II*)* ions detection* To detect labile Fe^2+^ ions, the cells were incubated with *FeRhoNox*™-1 probe (Goryo Chemical) according to the vendor’s protocol. The cells were stained with DAPI to exclude the DAPI + dead cells.

*Lipid peroxidation detection* Lipid Peroxidation Sensor BODIPY™ 581/591 C11 (Invitrogen) was used as an indicator of lipid oxidation. The cells were stained and analyzed according to the manufacturer’s instructions. Dead cells were excluded from analysis by DAPI staining.

*Reduced glutathione *(*GSH*)* detection* ThiolTracker Probe (Invitrogen) was used to detect the level of GSH. The cells were stained with DAPI to exclude the non-viable cells.

*Annexin V/propidium iodide *(*PI*)* staining* The ratio of dead and apoptotic cells was analyzed by an annexin V/PI staining. The cells were trypsinized, washed with annexin V binding buffer (140 mM NaCl, 2.5 mM CaCl_2_ in 10 mM HEPES, pH 7.4), and incubated with annexin V—APC (1% v/v; Exbio) in annexin V binding buffer and PI (100 μg/ml) for 20 min on ice. Then cells were washed, resuspended in annexin V binding buffer and the ratio of viable (annexin V−/PI−), early apoptotic cells (annexin V + /PI−), late apoptotic/necrotic (annexin V + /PI +) and dead (annexin V−/PI +) cells was determined.

All flow cytometry analyzes were performed with LSRFortessa flow cytometry analyzer (BD Biosciences) and analyzed in FlowJo version (v.10.0.7). Gating strategies are presented in supplementary figures (Fig. S1 and Fig. S2).

### Scratch-wound assay

hAdv cells were seeded at density of 10 000 cells per well of a 96-well plate. At 100% confluence, uniform scratch lines (0.9 mm width) were created using a scratcher (NanoEntek). The images of the scratch were taken immediately after wounding (0 h) and 4 h later. The wound closure areas (scratch area at time 0—cell-free area at time 4 h × 100%) were measured with ImageJ (Software 1.48q, Rayne Rasband, National Institutes of Health, USA).

### Transfection with small interfering RNA

The hAdv cells cultured in 24-well plates were transfected with 10 pmol of Silencer Select siRNAs (Thermo Fisher Scientific), either negative control siRNA No. 1 (siMock) or siRNA targeting human GDF-15 (siGDF-15, ID 147358), using Lipofectamine RNAiMAX reagent (Thermo Fisher Scientific) according to manufacturer’s instructions. The medium was changed after 24 h and the cells were analyzed after 72 h.

### EdU Cell Proliferation assay

Proliferation of hAdv cells was assessed with Click-iT™ Plus EdU Cell Proliferation Kit for Imaging, Alexa Fluor™ 594 dye (Invitrogen) according to manufacturer’s protocol. Briefly, hAdv cells were cultured in 24-well plates in the presence of GDF-15 or CM from control or senescent HAEC. After 48 h, the modified thymidine analogue EdU (5-ethynyl-2′-deoxyuridine) was added to the cells for 1 h. Then the cells were fixed, permeabilized and incubated with Click-iT® Plus reaction cocktail containing Alexa Fluor®594 picolyl azide. After 30 min the cells’ nuclei were stained with Hoechst® 33,342. Images acquisition was carried out using a Nikon Eclipse TS100 microscope.

### GDF-15 neutralization

The HAEC conditioned media were incubated for 1 h with 0.625–20 μg/ml of GDF-15-neutralizing antibody (Visugromab, CTL-002, MedChem Express). The concentration of 20 μg/ml of Visugromab was chosen as an effective one. In some experiments, Visugromab at the same 20 μg/ml concentration, was added directly to hAdv culture medium.

### Statistical analysis

All data are presented as mean ± standard deviation (SD) of three independent experiments using cells derived from a single donor. Statistical analyses were performed with GraphPad Prism (GraphPadSoftware Inc., San Diego, CA). Statistically significant differences between groups were determined using student’s *t*-test, if not stated otherwise, and a value of *p* < 0.05 was considered statistically significant.

## Results

### Oxidative stress affects senescence-associated proteins and pathways in endothelial cells

**Fig. 1:**
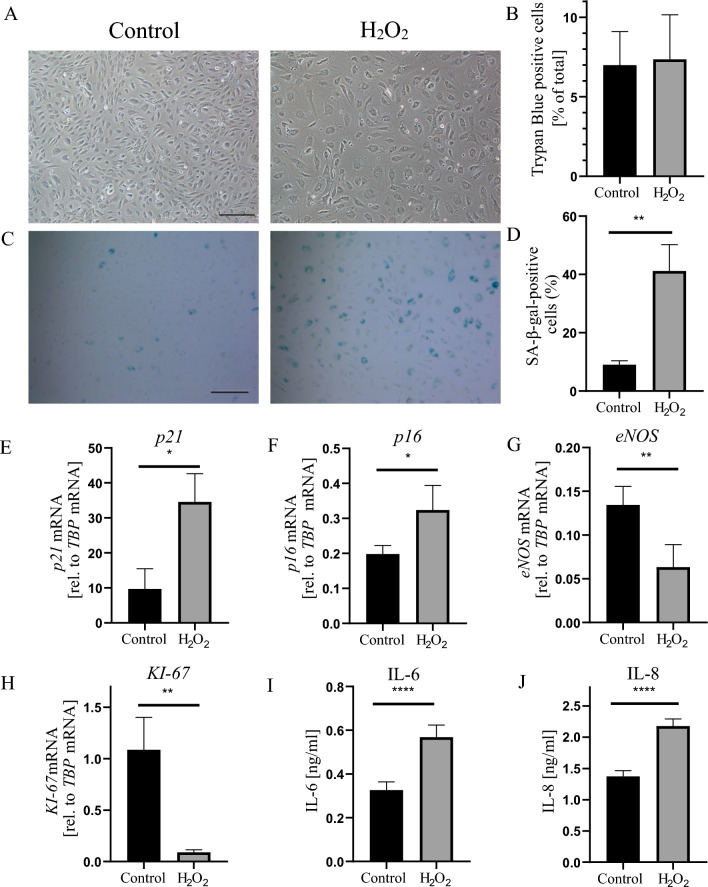
Hydrogen peroxide induces premature senescence of HAEC. **A** Representative phase-contrast microscopy pictures of control and H_2_O_2_-treated HAEC. **B** HAEC viability determined with trypan blue staining. **C** Representative bright field microscopy pictures of SA-β-gal staining in control and H_2_O_2_-treated HAEC. **D** Graph showing the percentage of SA-β-gal-positive cells. Relative mRNA levels of *p21* **E** and *p16* **F**, *eNOS* **G** and *Ki-67* **H** determined by RT-qPCR. Level of secreted IL-6 **I** and IL-8 J measured in CM by ELISA. Results are presented as mean ± SD (*p < 0.05, **p < 0.01, ***p < 0.001, ****p < 0.0001). Scale bar: 50 μm

Hydrogen peroxide, frequently used as an experimental source of oxygen-derived free radicals, was previously shown to effectively induce premature cell senescence [23–25]. Here, we used 75 μM concentration of H_2_O_2_ to induce premature senescence of HAEC cells. Higher concentrations of H_2_O_2_ (150 µM and 300 µM) decreased cell viability (Fig. S3A, B), and did not result in the increased number of SA-β-gal-positive senescent cells (Fig. S3C). After 72 h of culture (first 24 h in the presence of 75 µM H_2_O_2_) the microscopic examination revealed altered HAEC phenotype characterized by enlarged and flattened cell morphology (Fig. 1A). Trypan blue staining (Fig. 1B) and MTT assay (Fig. S3B) showed no evidence of increased cell death following the applied 75 µM concentration of H_2_O_2_. Next, the SA-β-gal activity was assessed as a biochemical marker of cellular senescence. As shown in Fig. 1C and D, administration of H_2_O_2_ markedly increased the number of SA-β-gal-positive cells, when compared to control HAEC. In addition, senescence of HAEC exposed to oxidative stress was validated by increased expression of cell cycle inhibitors—*CDKN1A* (*p21*, Fig. 1E) and *p16INK4a* (*p16*, Fig. 1F). In contrast, endothelial marker *eNOS* and proliferation marker *Ki-67* were downregulated (Fig. 1G and H, respectively) indicating impaired endothelial function and proliferative capacity. Increased expression of CDKN1A (p21) together with increased phosphorylated histone H2AX (phospho-H2AX), and decreased Ki-67 were confirmed on protein level (Fig. S3D). Finally, in H_2_O_2_-treated HAEC we observed significantly increased secretion of IL-6 (Fig. 1I) and IL-8 (Fig. 1J), two important SASP components [26].

**Fig. 2:**
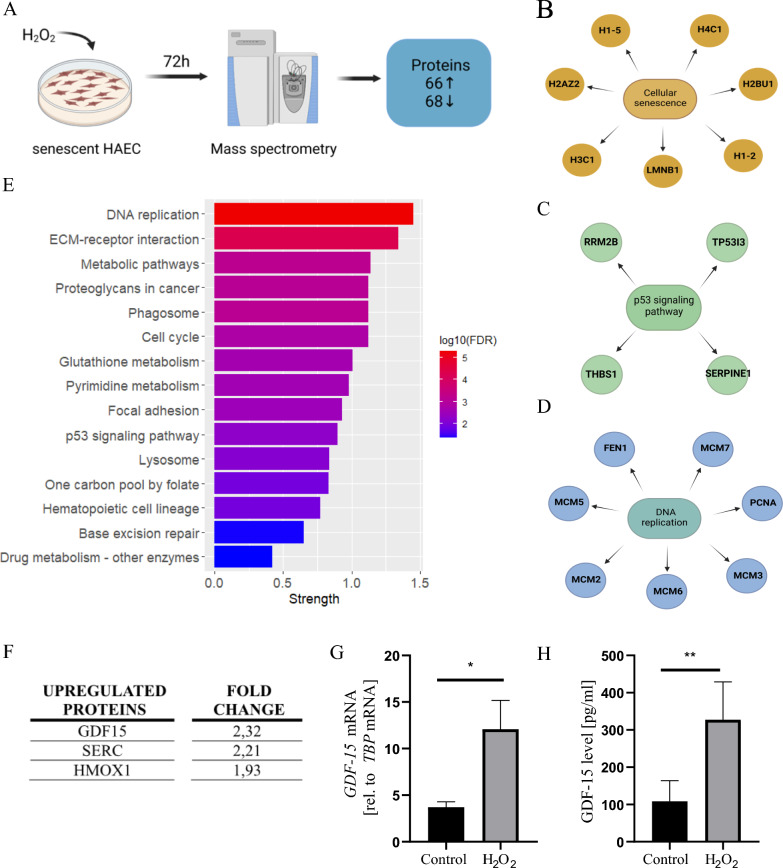
Extensive proteome changes in prematurely senescent HAEC. **A** Schematic depiction of the experiment. **B** Cellular senescence pathway (Reactome) enriched in senescent HAEC. **C** p53 signaling pathway (KEGG) enriched for senescent HAEC. **D** DNA replication pathway (KEGG) enriched for senescent HAEC. **E** The significantly enriched KEGG pathways of differentially expressed proteins in senescent HAEC. The x-axis represents the strength (log10(observed/expected)) of enriched pathways, whereas the color denotes log of the false discovery rate (FDR). **F** The top 3 upregulated proteins in senescent HAEC vs control HAEC identified in LC–MS/MS proteomic analysis. **G** *GDF-15* mRNA level measured by RT-qPCR. **H** GDF-15 protein level measured in CM by ELISA. Results are presented as mean ± SD (*p < 0.05, **p < 0.01)

After confirming the senescent phenotype of HAEC with the commonly used biochemical and molecular markers, we applied LC–MS/MS analysis (Fig. 2A) to better characterize the proteome of these cells. Proteomic profiling identified 134 significantly changed proteins. Among these, 66 proteins were upregulated and 68 were downregulated in senescent HAEC (Table S1). The results of the KEGG and Reactome pathway enrichment analysis revealed an association of the differentially expressed proteins in senescence-related signaling pathways: cellular senescence (Fig. 2B), p53 signaling (Fig. 2C) and DNA replication (Fig. 2D). Interestingly, one of the p53 targets, a profibrotic cytokine SERPINE1 (plasminogen activator inhibitor type-1; PAI-1), has been previously described as SASP component [27] capable of inducing paracrine senescence [28]. Other differentially expressed proteins were associated with base excision repair and cell cycle (Fig. 2E), what can be related to oxidative DNA damage and cell cycle arrest upon oxidative stress. In addition, phagosome, glutathione metabolism and other metabolic pathways were significantly enriched (Fig. 2E) demonstrating loss of protein homeostasis. Changes in proteins involved in ECM-receptor interaction and focal adhesion (Fig. 2E) may indicate loss of integrity and alterations in endothelial functional phenotype. In addition, our proteomic analysis revealed that the top 3 proteins upregulated in senescent HAEC (Fig. 2F, Table S1) are involved in cell survival. Among these, we found stress-responsive growth differentiation factor 15 (GDF-15), a pro-proliferative phosphoserine aminotransferase 1 (SERC/PSAT1), and redox-regulated enzyme heme oxygenase-1 (HMOX1). We confirmed an increased expression of GDF-15 in H_2_O_2_-treated senescent HAEC at the mRNA level (Fig. 2G) and via its increased secretion to the cell culture medium (Fig. 2H).

### Proteomic changes in hAdv cells treated with CM from senescent HAEC

**Fig. 3:**
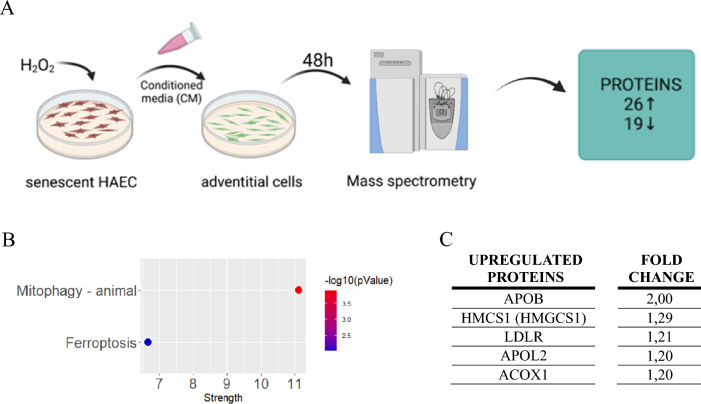
Proteomic changes in hAdv cells treated with CM from senescent HAEC. **A** Schematic depiction of the experiment. **B** The significantly enriched KEGG pathways of upregulated and downregulated proteins in hAdv cells treated with CM from senescent HAEC vs. CM from control HAEC. The x-axis represents the strength (Log10(observed/expected)) of enriched pathways, whereas the color denotes –log10(pValue). **C** The upregulated proteins involved in lipids uptake and metabolism in hAdv cells treated with CM from senescent HAEC vs CM from control HAEC identified in LC–MS/MS analysis

Endothelial cells undergoing senescence during normal aging, may exert paracrine effects on other blood vessels-forming cells [29]. Adventitial cells (particularly fibroblasts) are often the first cells in the vascular wall to become activated in response to various stimuli and can actively participate in arterial remodeling or neointimal formation [3]. Here, we wanted to analyze the effect of endothelial cell-derived SASP on adventitial fibroblasts. The cells were exposed to CM derived from either control or senescent HAEC, in which senescence was induced via addition of H_2_O_2_ 72 h earlier (Fig. 3A). CM from senescent HAEC did not change the migration (Fig. S4A, B) and slightly increased the proliferation rate of hAdv cells (Fig. S4C, D). Quantitative proteomics identified 26 upregulated and 19 downregulated proteins in hAdv cells cultured in the presence of CM from senescent HAEC (Table S2). KEGG pathway analysis of differentially expressed proteins in hAdv cells treated with SASP CM revealed association with pathways related to the process of selective degradation of mitochondria (mitophagy) and ferroptosis—a regulated form of cell death associated with accumulation of toxic lipid peroxides (Fig. 3B). Apart from these, among the upregulated proteins not linked to the above-mentioned KEGG pathways, we identified proteins commonly known to be associated with lipids uptake or turnover – apolipoprotein B (APOB), hydroxymethylglutaryl-CoA synthase (HMCS1/HMGCS1), low-density lipoprotein receptor (LDLR), apolipoprotein L2 (APOL2), and peroxisomal acyl-coenzyme A oxidase 1 (ACOX1; Fig. 3C).

### GDF-15 increases the expression of genes involved in iron storage and ferroptosis

**Fig. 4:**
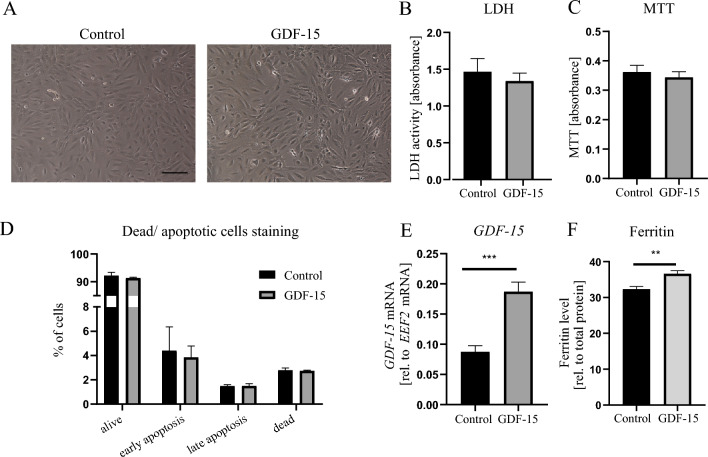
Effects of rhGDF-15 on hAdv cells in basal conditions. **A** The morphology of hAdv cells 48 h upon stimulation. **B** LDH activity in conditioned media. **C** MTT assay. **D** The ratio of alive, dead, early- and late-apoptotic hAdv cells assessed by flow cytometry after Annexin V and PI staining. **E** Relative mRNA level of *GDF-15* determined by RT-qPCR. **F** Ferritin ELISA in cell lysates upon rhGDF-15 stimulation. Bars represent mean ± SD (** p < 0.01, *** p < 0.001). Scale bar: 50 μm

Paracrine effects of senescent cells are mediated via SASP [11, 12]. Our proteomic analysis showed that a secretory factor GDF-15 was the top upregulated protein in senescent HAEC (Table S1). Moreover, ELISA assay confirmed an increased release of this protein from senescent HAEC (Fig. 2H). Thus, in the next steps, we wanted to investigate if GDF-15 might be responsible for some of the observed paracrine effects of CM from senescent HAEC on adventitial cells. According to the performed ELISA assay, the concentration of GDF-15 in CM from senescent HAEC (which was used for treatment of hAdv cells) was approx. 327 ± 102 pg/ml/48 h (Fig. 2H). However, the effective in vitro concentrations of recombinant GDF-15 are much higher—frequently around 200 ng/ml [30, 31]. The morphology of hAdv cells treated with this concentration (200 ng/ml) of rhGDF-15 indicated no signs of cytotoxicity (Fig. 4A). The hAdv cell viability measured with MTT assay (Fig. 4B) and cytotoxicity detected with LDH activity assay (Fig. 4C) remained unchanged upon treatment with 200 ng/ml rhGDF-15. The quantitative analysis of necrotic and apoptotic cells stained with annexin V and PI showed an equal abundance of alive, early or late apoptotic and dead cells in control and rhGDF-15-treated hAdv cells (Fig. 4D). At the mRNA level, we observed upregulation of GDF-15 expression in hAdv cells treated with rhGDF-15 (Fig. 4E). This is consistent with other data demonstrating that GDF-15 induces its own expression [31].

No changes in hAdv cells migration (Fig. S5A, B) were observed upon rhGDF-15 stimulation. The proliferation rate was slightly increased upon rhGDF-15 stimulation (Fig. S5C, B), resembling the effect of senescent HAEC-derived CM (Fig. S4C, D). Thus, in order to examine whether the effect of increased hAdv proliferation upon senescent CM stimulation depends on senescent endothelial cells-derived GDF-15, in the next series of experiments we used Visugromab, a GDF-15 neutralizing antibody. First, we tested different concentrations of Visugromab (0.625–20 μg/ml) in senescent HAEC-derived CM (Fig. S6A). The concentration of 20 μg/ml was chosen as the most effective for GDF-15 neutralization. However, addition of Visugromab to senescent CM did not affect hAdv cells proliferation (Fig. S6B) indicating that the stimulatory effect of senescent endothelial cells-derived CM on hAdv cells proliferation is most probably mediated by some other than GDF-15 SASP component. Interestingly, addition of Visugromab directly to hAdv cells culture medium resulted in decreased proliferation rate of the cells (Fig. S6C) indicating involvement of GDF-15 in autocrine regulation of this process.

One of the KEGG pathways enriched in hAdv cells cultured in the presence of CM from senescent HAEC was mitophagy (Fig. 3B). In order to check whether GDF-15 might be involved in this process, we used the MitoTracker fluorescent dye (ThermoFisher Scientific) to label mitochondria in the control and GDF-15-treated hAdv cells. However, there was no difference between both groups (data not shown). Thus, we next focused on the second identified KEGG pathway. The proteomic analysis performed in hAdv cells treated with SASP CM demonstrated upregulation of proteins involved in iron storage and ferroptosis, i.e. two subunits of the main intracellular iron binding protein: ferritin heavy chain (FRIH/FTH1; Table S2) and ferritin light chain (FRIL/FTL; Table S2), as well as ferroptosis-related microtubule-associated protein 1 light chain 3 beta 2 (MP3B2/MAP1LC3B2; Table S2). These changes suggested increased protection of hAdv exposed to SASP CM against oxidative damage. Interestingly, we observed that rhGDF-15, similarly to CM from senescent HAEC, increased the level of ferritin in hAdv cells (Fig. 4F).

**Fig. 5:**
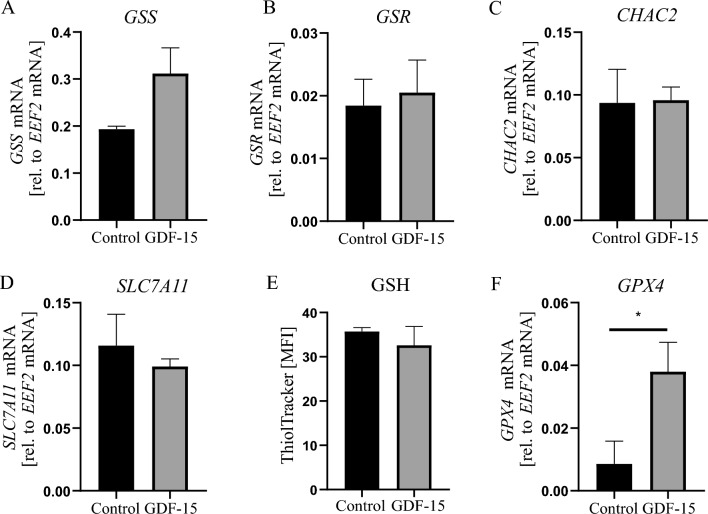
Effect of rhGDF-15 on glutathione homeostasis in hAdv cells. Relative mRNA levels of **A** *GSS*, **B** *GSR*, **C** *CHAC2*, **D** *SLC7A11*. **E** Glutathione level in cell lysates detected by flow cytometry with ThiolTracker probe. **H** Relative mRNA level of *GPX4*. Bars represent mean ± SD (*p < 0.05, ***p < 0.001)

Next, we analyzed the expression of selected genes providing protection against oxidative stress, mainly the genes responsible for maintaining the balance of reduced glutathione (GSH), in hAdv cells treated with rhGDF-15. The mRNA levels of glutathione synthetase (*GSS*; Fig. 5A), glutathione-disulfide reductase (*GSR*; Fig. 5B), glutathione specific gamma-glutamylcyclotransferase 2 (*CHAC2*; Fig. 5 C) and, a main transporter of cysteine, solute carrier family 7 member 11 (*SLC7A11*, Fig. 5D) were not affected by rhGDF-15 treatment. Moreover, the level of GSH detected with ThiolTracker probe also did not differ between control and rhGDF-15-treated hAdv cells (Fig. 5F). On the other hand, the expression of ferroptosis-related hydroperoxide scavenger glutathione peroxidase 4 (*GPX4*) [32], was increased in hAdv cells treated with rhGDF-15 (Fig. 5F).

### Stimulation with GDF-15 does not affect erastin-induced hAdv cell death

**Fig. 6:**
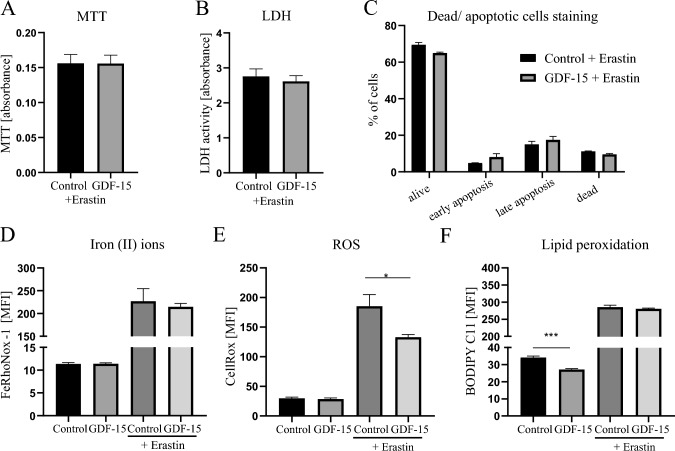
Effect of rhGDF-15 on erastin-induced cell death in hAdv cells. **A** MTT assay. **B** LDH assay. **C** The percentage of viable, apoptotic and dead hAdv cells treated with erastin in the presence or absence (control) of rhGDF-15 (AnnexinV and PI staining). **D** Fe^2+^ level in cell lysates detected with FeRhoNox-1 probe. **E** ROS level in cell lysates detected with CellRox Deep Red Reagent. **F** Lipid peroxidation level detected with BODIPY™ 581/591 C11 probe. Bars represent mean ± SD (*p < 0.05)

Since rhGDF-15 increased the level of ferritin (Fig. 4F) and induced the expression of *GPX4* (Fig. 5F) in hAdv cells, we next wanted to determine if this growth factor might confer protection against erastin-induced ferroptotic cell death. Erastin is capable of initiating ferroptosis via prevention of the synthesis of antioxidant glutathione in the cell. To investigate the cellular cytotoxicity of erastin, hAdv cells were treated for 24 h with different concentrations (0.5, 1, 2, and 5 µM) of erastin. We found that the 2 µM concentration was already very toxic and caused almost complete cell death (Fig. S7A, B). According to the MTT assay, 1 µM concentration resulted in an approx. 30% reduction of cell viability (Fig. S7B) and was chosen as optimal for further experiments. Regardless of whether erastin-treated hAdv cells were pretreated or not with rhGDF-15, there were no differences in cell viability (Fig. 6A) and cytotoxicity (Fig. 6B). The quantitative analysis of necrotic and apoptotic cells stained with annexin V and PI also showed no difference in the percentage of early or late apoptotic and dead cells pretreated or not with rhGDF-15 before addition of erastin (Fig. 6C). There was also no difference in the level of iron Fe^2+^ ions between hAdv cells treated with erastin and erastin + rhGDF-15 (Fig. 6D). Interestingly, we observed a significant decrease in erastin-induced ROS level detected with CellRox dye in rhGDF-15-treated hAdv cells (Fig. 6E), what might suggest the activation of some antioxidant protective mechanisms by GDF-15. In addition, the signal from BODIPY 581/591 C11, a fluorescent lipid peroxidation sensor, was reduced by rhGDF-15 in hAdv cells cultured in basal conditions (Fig. 6F), but not in cells treated with erastin (Fig. 6F).

### Silencing of endogenous GDF-15 alleviates ferroptosis of hAdv cells

**Fig. 7:**
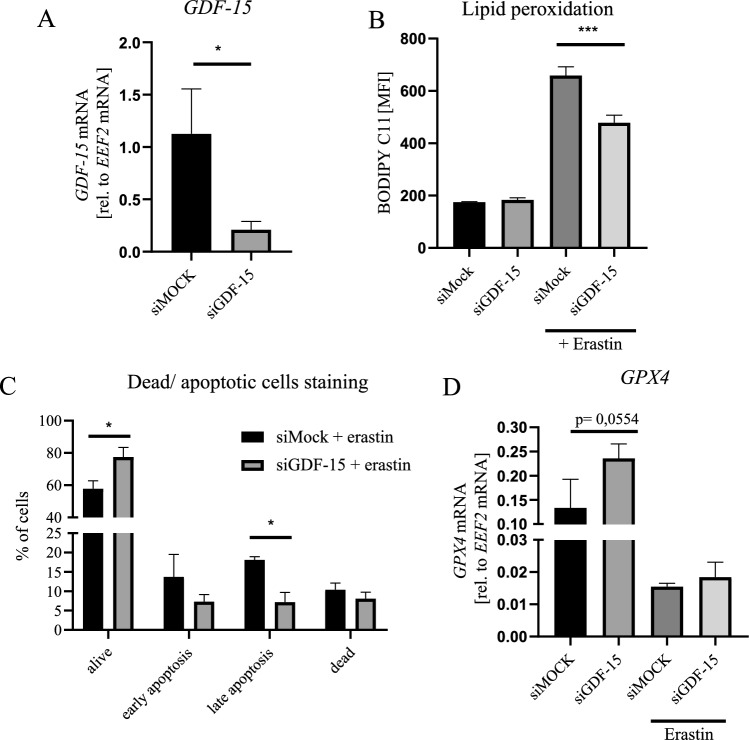
Effect of siGDF-15 on erastin-induced cell death in hAdv cells. **A** Relative mRNA level of *GDF-15*. **B** Lipid peroxidation level detected with BODIPY™ 581/591 C11 probe. **C** The percentage of viable, apoptotic and dead hAdv cells treated with erastin following siMock or siGDF-15 transfection (AnnexinV and PI staining). **D** Relative mRNA level of *GPX4*. Bars represent mean ± SD (*p < 0.05)

In basal conditions, hAdv cells secrete relatively high amount of GDF-15 (536 ± 31 pg/ml/48 h) exceeding the amount detected in CM from control and senescent HAEC (Fig. 2H) used for hAdv stimulation. As demonstrated, addition of exogenous GDF-15 had no effect on survival of hAdv cells treated with erastin (Fig. 6C). Thus, next we wanted to check whether silencing of endogenous GDF-15 (Fig. 7A) could impact hAdv ferroptotic cell death. Decreasing GDF-15 expression with siRNA did not affect hAdv cells viability as compared to control cells transfected with siMock (Fig. S8). Next, at 48 h following the addition of siRNA against *GDF-15* the cells were treated with 1 µM concentration of erastin. Interestingly, silencing of GDF-15 decreased lipid peroxidation (Fig. 7B) and improved survival (higher percentage of viable cells and lower percentage of late apoptotic cells; Fig. 7C) of hAdv cells exposed to erastin treatment. However, *GPX4* expression did not differ significantly between siMock and siGDF-15, neither in basal conditions nor upon erastin treatment (Fig. 7D), indicating that GPX4 is not involved in this protective effect of GDF-15.

## Discussion

Senescent cells accumulate with age and in age-associated diseases [5]. The well-known contributor to endothelial cell senescence and vascular aging is oxidative stress caused by cytotoxic levels of ROS. Here, we used H_2_O_2_ to induce premature senescence of HAEC cells in vitro. In order to better characterize the proteomic changes in oxidative stress-induced endothelial cell senescence, we performed mass spectrometry analysis. We found that the differentially expressed proteins were involved not only in cell senescence, cell cycle and metabolism, but also in ECM-receptor interaction and focal adhesion. Interestingly, a recent study by Grandy et al*.* demonstrated remodeling of the focal adhesion complex in H_2_O_2_‑induced senescence of NIH 3T3 mouse fibroblasts and suggested that such alterations may lead to disturbed mechanosensing and change the senescent cell behavior [33].

Clearance of senescent cells with either genetic or pharmacologic approaches was shown to delay or inhibit several age-associated disorders in mice [34]. Senescent cells activate survival mechanisms and become resistant to apoptotic cell death [35]. Senolytic drugs (e.g. dasatinib, quercetin, navitoclax) promote apoptosis of senescent cells by temporarily disabling the pro-survival pathways. However, the currently available senolytics lack specificity, suggesting that a targeted approach could be more clinically relevant. Our proteomic analysis revealed that the top 3 proteins upregulated in senescent HAEC (Fig. 2F, Table S1) are involved in cell survival. Among these, we found stress-responsive growth differentiation factor 15 (GDF-15) [36], a pro-proliferative phosphoserine aminotransferase 1 (SERC/PSAT1) [37], and redox-regulated enzyme heme oxygenase-1 (HMOX1) [38]. Interestingly, HMOX1 was recently identified as a common senolytic target in two senescent cell types—satellite cells and fibro-adipogenic progenitors, in which senescence was induced via doxorubicin treatment [39]. Limbad et al*.* also demonstrated that shRNA-mediated knockdown of *Hmox1* significantly decreased viability of senescent cells, while non-senescent cells were not affected [39]. Our data from senescent HAECs also indicate that HMOX1 may be considered as an interesting senolytic target.

There is mounting evidence that senescent cells exert multiple paracrine effects on neighboring cells, including induction of paracrine senescence in normal cells [40] and stimulation of proliferation of transformed fibroblasts and tumor cells [26, 41, 42]. Accordingly, we observed increased proliferation of adventitial fibroblasts exposed to endothelial cell-derived SASP. As a source of chronic inflammation and some plaque instability factors, SASP was also shown to be involved in the pathogenesis and development of atherosclerosis [12]. Our KEGG pathway analysis of differentially expressed proteins in hAdv cells treated with senescent HAEC-derived CM revealed association with pathways related to mitophagy and ferroptosis—a form of cell death due to accumulation of toxic lipid peroxides. In addition, in hAdv cells exposed to CM from senescent HAEC we identified several proteins associated with lipids uptake or turnover—APOB, HMCS1/HMGCS1, LDLR, APOL2, and ACOX1. The top upregulated protein, APOB, is the major component of low-density lipoproteins (LDL) providing binding sites for LDL receptor (LDLR). APOB selectively interacts with the elements of ECM, such as collagen and proteoglycans, enabling endocytosis of LDL particles [43]. Moreover, the protein levels of APOB were shown to be significantly higher in late senescent than in young human dermal fibroblasts [44], what additionally links this protein to cellular senescence and age-related diseases. Fibroblasts of the arterial wall mostly reside in the adventitial layer [1]. The high plasticity of these cells supported by their involvement in fibrosis, inflammation, and angiogenesis, makes them an important player in atherosclerosis and vascular aging [45]. Recent single-cell RNA sequencing (scRNA-seq) data underline the transcriptional heterogeneity of arterial fibroblasts and the influence of pathologic environments, such as lipid levels or aging, on fibroblasts differentiation [46].

Senescent cells are also often characterized by and exert their paracrine biological effects via SASP components [11, 12]. Nowadays, it is well understood that there is no uniform SASP, because its composition and effects can be affected by various factors [47]. There is currently a great interest in SASP profiles, which might serve as senescence biomarkers in human plasma or other biofluids and help to assess the efficacy of senescence-targeted therapies. In line with this, Basisty et al*.* recently defined in senescent culture cells a core SASP—common SASP components regardless of the cell type, senescence inducer, micro-environment, or time, which passed from addition of senescence trigger. Among the core SASP proteins they distinguished GDF-15, stanniocalcin 1 (STC1), SERPINE1/PAI-1, and MMP1 [11]. Importantly, these factors are also reported to be significantly increased among the plasma markers of ageing in humans [11]. Our proteomic analysis in senescent HAEC confirms these observations, as we observed significant induction of two of the above mentioned secretory proteins: PAI-1 and the top upregulated in our analysis GDF-15.

GDF-15 is a member of the transforming growth factor (TGF)-β superfamily. As a stress-induced cytokine, the expression and secretion of GDF-15 can be strongly induced by cellular stress [36]. GDF-15 concentrations in peripheral blood are an established predictive biomarker of all-cause mortality and of adverse cardiovascular events [48]. Moreover, GDF-15 is considered a promising candidate biomarker of cellular senescence, as its level overlaps with aging markers in human plasma [11]. The loss of GDF-15 was recently shown to induce mitochondrial dysfunction and premature senescence in dermal fibroblasts [49]. It was also demonstrated that GDF-15 produced by senescent endothelial cells exerts paracrine effects on non-senescent cells [50]. Thus, we wanted to investigate if GDF-15 might be responsible for some of the observed paracrine effects of CM from senescent HAEC on hAdv cells. Similarly to endothelial cell-derived SASP, rhGDF-15 induced proliferation of the adventitial fibroblast cell line. However, neutralization of GDF-15 did not prevent the stimulatory effect of senescent HAEC-derived CM on hAdv cells proliferation indicating that this effect of CM is most probably mediated by some other than GDF-15 SASP component.

Moreover, we observed that rhGDF-15 increased the level of ferritin—a major cellular iron storage protein and the expression of *GPX4* in hAdv cells. GPX4 activity maintains lipid homeostasis and protects cells against the accumulation of toxic lipid ROS and the onset of iron-dependent oxidative cell death termed ferroptosis [32]. It was shown that ferroptosis of macrophages in advanced plaques accelerates atherosclerosis and can lead to plaque destabilization [51]. On the contrary, inhibition of ferroptosis was demonstrated to alleviate atherosclerosis [52]. Increased expression of GPX4 was able to inhibit the development of atherosclerosis by decreasing lipid peroxidation and inhibiting the sensitivity of vascular cells to oxidized lipids [53]. Up to date, only a few studies demonstrated that GDF-15 is linked to ferroptosis. GDF-15 knockdown was shown to promote erastin-induced ferroptosis in human gastric cancer cell line MGC803 [54]. Contrary to this, a more recent paper showed that downregulation of GDF-15 in clear cell renal cell carcinoma suppresses ferroptosis [55]. Another study demonstrated that recombinant GDF-15 alleviated spinal cord injury-induced neuronal ferroptosis by regulating the p62-Keap1-Nrf2 signaling pathway [56]. Thus, the effect of GDF-15 on ferroptosis is not clear. Here, we wanted to check if rhGDF-15, in our hands causing upregulation of ferritin and *GPX4*, might protect hAdv cells from ferroptotic cell death. Although affecting some ferroptosis-related factors and decreasing oxidative stress, treatment with GDF-15 had no effect on erastin-induced cell death of the analyzed adventitial fibroblast cell line. Interestingly, silencing of GDF-15 with siRNA protected hAdv cells against erastin-induced cell death. The mechanism responsible for this observation remains to be elucidated.

In summary, we have presented here a detailed characteristics of oxidative stress-induced human aortic endothelial cells senescence. We found that, in in vitro settings, such prematurely senescent aortic endothelial cells affect, in a paracrine manner, the proteome of adventitial fibroblasts. It is proposed that in in vivo conditions, such signaling can occur either via the *vasa vasorum* or by direct diffusion. Our proteomic analysis showed that the majority of differentially expressed proteins in hAdv cells treated with CM from senescent endothelial cells were involved in lipids uptake and metabolism, mitophagy and susceptibility to ferroptosis. We have also shown that GDF-15, an important SASP component, affects some elements of the ferroptotic pathway, although an exogenous GDF-15 does not influence erastin-induced cell death of the analyzed adventitial fibroblast cell line. On the other hand, the influence of endogenous GDF-15 on the sensitivity of hAdv cells to ferroptotic stimuli was significant. It should be noted that the specific outcomes can vary depending on the cellular context, as GDF-15 may have diverse effects on different cell types and in different microenvironments. In the future, it might be worth to reproduce these findings in primary adventitial fibroblasts or in an appropriate in vivo model. We believe that our findings provide a better understanding of the biology of senescent cells and can be of importance for potential therapeutic strategies targeting cell senescence or ferroptosis to alleviate neointima and atherosclerotic plaque formation.

## Supplementary Information

Below is the link to the electronic supplementary material.

Supplementary file1 (DOCX 2996 kb)

## Data Availability

The mass spectrometry data were deposited to the ProteomeXchange Consortium via the MassIVE repository with the dataset identifier PXD049353.
