# Supplementary material for: Senescence of endothelial cells promotes phenotypic changes in adventitial fibroblasts: possible implications for vascular aging
Source: Mol Cell Biochem. 2024 May 14;480(2):1027–43. doi: 10.1007/s11010-024-05028-7 (PMC11835997; doi:10.1007/s11010-024-05028-7)
Supplement: Supplementary file 1 — Supplementary file1 (DOCX 2996 kb) [file 11010_2024_5028_MOESM1_ESM.docx]

Table S1. Proteome changes in senescent versus control HAEC. Differentially expressed proteins and their fold change (FC).

| **Protein** | **FC** | **Protein** | **FC** |  | **Protein** | **FC** | **Protein** | **FC** |
| --- | --- | --- | --- | --- | --- | --- | --- | --- |
| **MCM2** | -2,62 | **SMCA5** | -1,44 |  | **GDF15** | 2,32 | **VA0D1** | 1,32 |
| **H12** | -2,56 | **MMRN1** | -1,43 |  | **SERC** | 2,21 | **FLNC** | 1,31 |
| **TYSY** | -2,50 | **RED** | -1,42 |  | **HMOX1** | 1,93 | **VAT1** | 1,31 |
| **MCM6** | -2,33 | **H4** | -1,41 |  | **PARP9** | 1,72 | **6PGD** | 1,30 |
| **MCM7** | -2,17 | **LMNB1** | -1,41 |  | **RIR2B** | 1,72 | **ITA5** | 1,30 |
| **MCM5** | -2,14 | **H31** | -1,41 |  | **AKA12** | 1,63 | **SPNS1** | 1,29 |
| **MCM3** | -2,10 | **PGBM** | -1,39 |  | **VPP1** | 1,59 | **STOM** | 1,29 |
| **SMC4** | -2,09 | **CBX5** | -1,38 |  | **TAGL** | 1,56 | **ITA2** | 1,29 |
| **DUT** | -2,00 | **RIR1** | -1,36 |  | **PVR** | 1,55 | **GARS** | 1,29 |
| **DYR** | -1,99 | **DDX5** | -1,36 |  | **NRCAM** | 1,52 | **CATB** | 1,28 |
| **H15** | -1,98 | **TCEA1** | -1,35 |  | **CADH2** | 1,51 | **GSDME** | 1,27 |
| **H1X** | -1,97 | **RS14** | -1,35 |  | **CMBL** | 1,48 | **RFIP5** | 1,27 |
| **CDK1** | -1,90 | **LAP2B** | -1,35 |  | **NPC1** | 1,46 | **MLP3B** | 1,27 |
| **DNJC9** | -1,85 | **TINAL** | -1,34 |  | **S12A9** | 1,44 | **GLGB** | 1,26 |
| **FEN1** | -1,80 | **PCNA** | -1,34 |  | **OCAD2** | 1,43 | **PTN1** | 1,26 |
| **HMGB3** | -1,78 | **SYAC** | -1,32 |  | **NEUA** | 1,43 | **COTL1** | 1,26 |
| **TSP1** | -1,78 | **NUCL** | -1,31 |  | **VAS1** | 1,43 | **OFUT2** | 1,26 |
| **H2B3B** | -1,68 | **GIMA7** | -1,30 |  | **PAI1** | 1,43 | **HYEP** | 1,26 |
| **VWF** | -1,67 | **CISD2** | -1,29 |  | **DGKA** | 1,43 | **NAGK** | 1,25 |
| **NASP** | -1,64 | **HCFC1** | -1,29 |  | **PLXA2** | 1,43 | **6PGL** | 1,24 |
| **FA98B** | -1,64 | **CYBP** | -1,29 |  | **AKAP2** | 1,42 | **AMPN** | 1,24 |
| **IMA1** | -1,62 | **E41L2** | -1,29 |  | **ADRO** | 1,42 | **MVP** | 1,24 |
| **LAP2A** | -1,60 | **P3H3** | -1,29 |  | **AHNK2** | 1,41 | **PLST** | 1,24 |
| **H2AV** | -1,59 | **IDI1** | -1,28 |  | **UCHL1** | 1,40 | **REEP5** | 1,23 |
| **DDX21** | -1,57 | **RTRAF** | -1,26 |  | **NMT2** | 1,37 | **NB5R3** | 1,23 |
| **HMCS1** | -1,57 | **ROA1** | -1,26 |  | **ANXA4** | 1,36 | **DPYL2** | 1,23 |
| **NHRF1** | -1,55 | **C1TC** | -1,25 |  | **CD44** | 1,35 | **ITAV** | 1,22 |
| **PSIP1** | -1,53 | **RHG18** | -1,24 |  | **ARRB1** | 1,35 | **RAP1A** | 1,22 |
| **FABP4** | -1,52 | **HNRPC** | -1,23 |  | **TIGAR** | 1,35 | **ITB3** | 1,21 |
| **PARP1** | -1,51 | **STAB1** | -1,23 |  | **KAD1** | 1,34 | **GSTO1** | 1,21 |
| **HPF1** | -1,49 | **ROA2** | -1,23 |  | **CLCB** | 1,33 | **GELS** | 1,21 |
| **MYG1** | -1,49 | **NAA50** | -1,23 |  | **GPI8** | 1,32 | **PNPH** | 1,21 |
| **PIR** | -1,46 | **DDX1** | -1,23 |  | **QORX** | 1,32 |  |  |
| **NUMA1** | -1,45 | **KPRB** | -1,20 |  | **GSTK1** | 1,32 |  |  |

Table S2. Proteomic analysis of senescent CM treated hAdv cells versus control CM treated hAdv cells. Differentially expressed proteins with fold change (FC) and p-value.

| **Protein** | **FC** | **p-value** |  | **Protein** | **FC** | **p-value** |
| --- | --- | --- | --- | --- | --- | --- |
| **APOB** | 2,00 | 0,008 |  | **OCTC** | -1,30 | 0,028 |
| **TAXB1** | 1,56 | 0,002 |  | **RM22** | -1,30 | 0,021 |
| **TSN3** | 1,55 | 0,034 |  | **FBLN3** | -1,29 | 0,001 |
| **CD320** | 1,53 | 0,032 |  | **THOC5** | -1,29 | 0,014 |
| **SC11C** | 1,48 | 0,029 |  | **NIPS2** | -1,28 | 0,042 |
| **CEGT** | 1,47 | 0,007 |  | **SAP3** | -1,27 | 0,042 |
| **CACO2** | 1,44 | 0,003 |  | **RM47** | -1,26 | 0,011 |
| **ITM2B** | 1,42 | 0,001 |  | **EMC3** | -1,26 | 0,020 |
| **FRIL** | 1,37 | 0,008 |  | **PPT1** | -1,25 | 0,023 |
| **SQSTM** | 1,33 | 0,005 |  | **CRLF3** | -1,25 | 0,049 |
| **MP3B2** | 1,30 | 0,018 |  | **CETN2** | -1,24 | 0,037 |
| **FRIH** | 1,30 | 0,000 |  | **CBWD1** | -1,22 | 0,042 |
| **GDN** | 1,29 | 0,006 |  | **PLBL2** | -1,22 | 0,016 |
| **HMCS1** | 1,29 | 0,018 |  | **STK39** | -1,21 | 0,019 |
| **S38A2** | 1,28 | 0,031 |  | **PPIE** | -1,21 | 0,010 |
| **A4** | 1,27 | 0,021 |  | **EXOS2** | -1,20 | 0,027 |
| **PPM1B** | 1,27 | 0,048 |  | **CO6A1** | -1,20 | 0,003 |
| **STX8** | 1,25 | 0,036 |  | **CATC** | -1,20 | 0,050 |
| **RB27B** | 1,25 | 0,027 |  | **NIPS1** | -1,20 | 0,037 |
| **ANTR2** | 1,22 | 0,016 |  |  |  |  |
| **MPZL1** | 1,22 | 0,025 |  |  |  |  |
| **GBRL2** | 1,22 | 0,037 |  |  |  |  |
| **LDLR** | 1,21 | 0,034 |  |  |  |  |
| **CTR9** | 1,21 | 0,032 |  |  |  |  |
| **APOL2** | 1,20 | 0,043 |  |  |  |  |
| **ACOX1** | 1,20 | 0,004 |  |  |  |  |


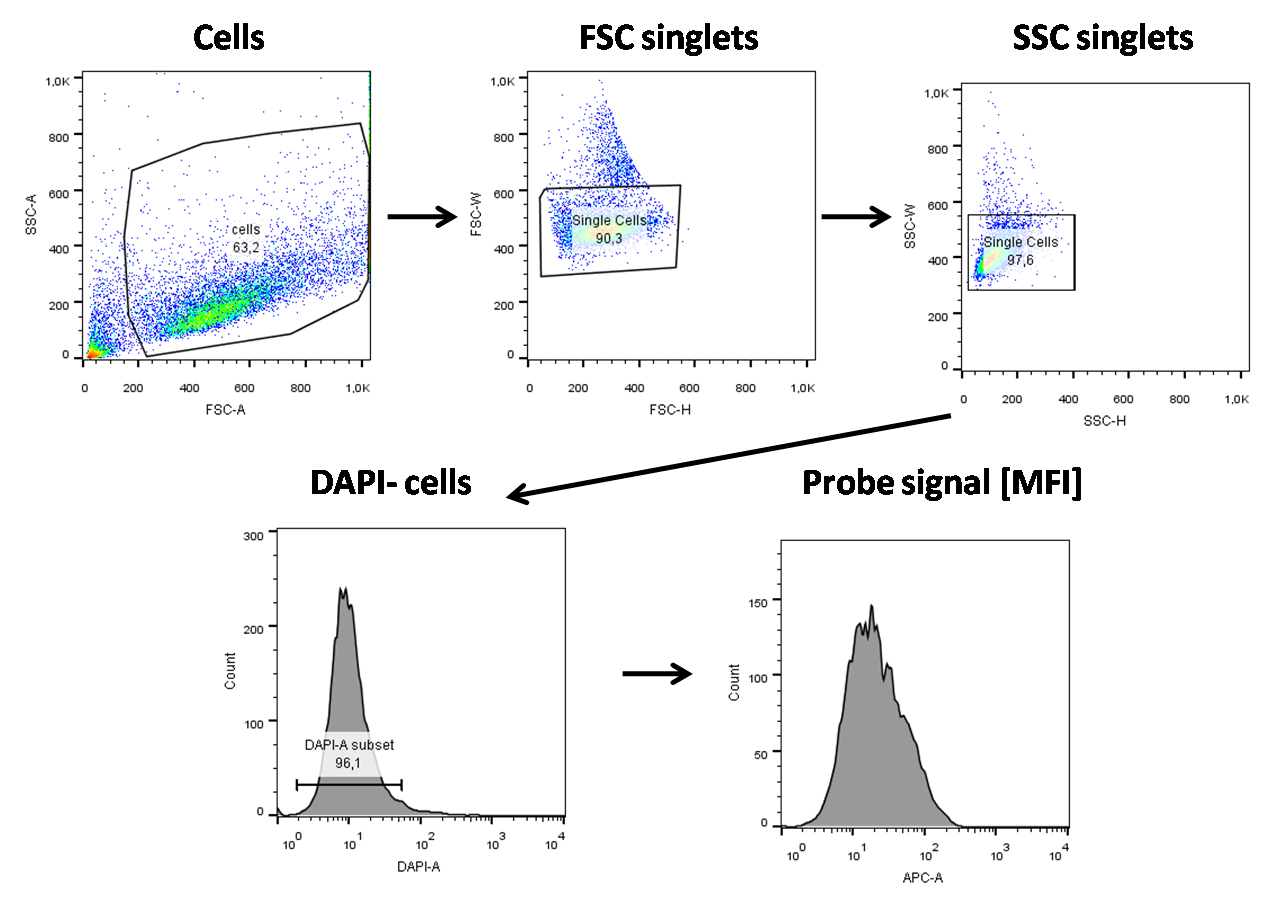


**Fig. S1** Example of gating strategy for flow cytometry analysis of GSH, ROS, Lipid peroxidation, iron (II) ions staining.


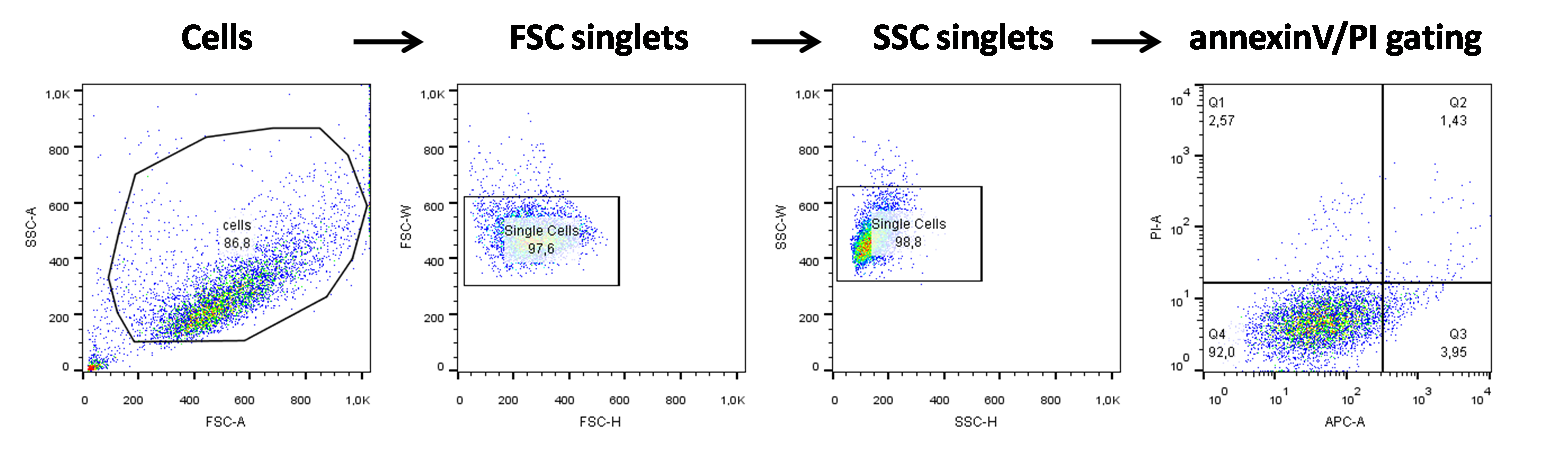


**Fig. S2** Example of gating strategy for annexin V/PI staining.


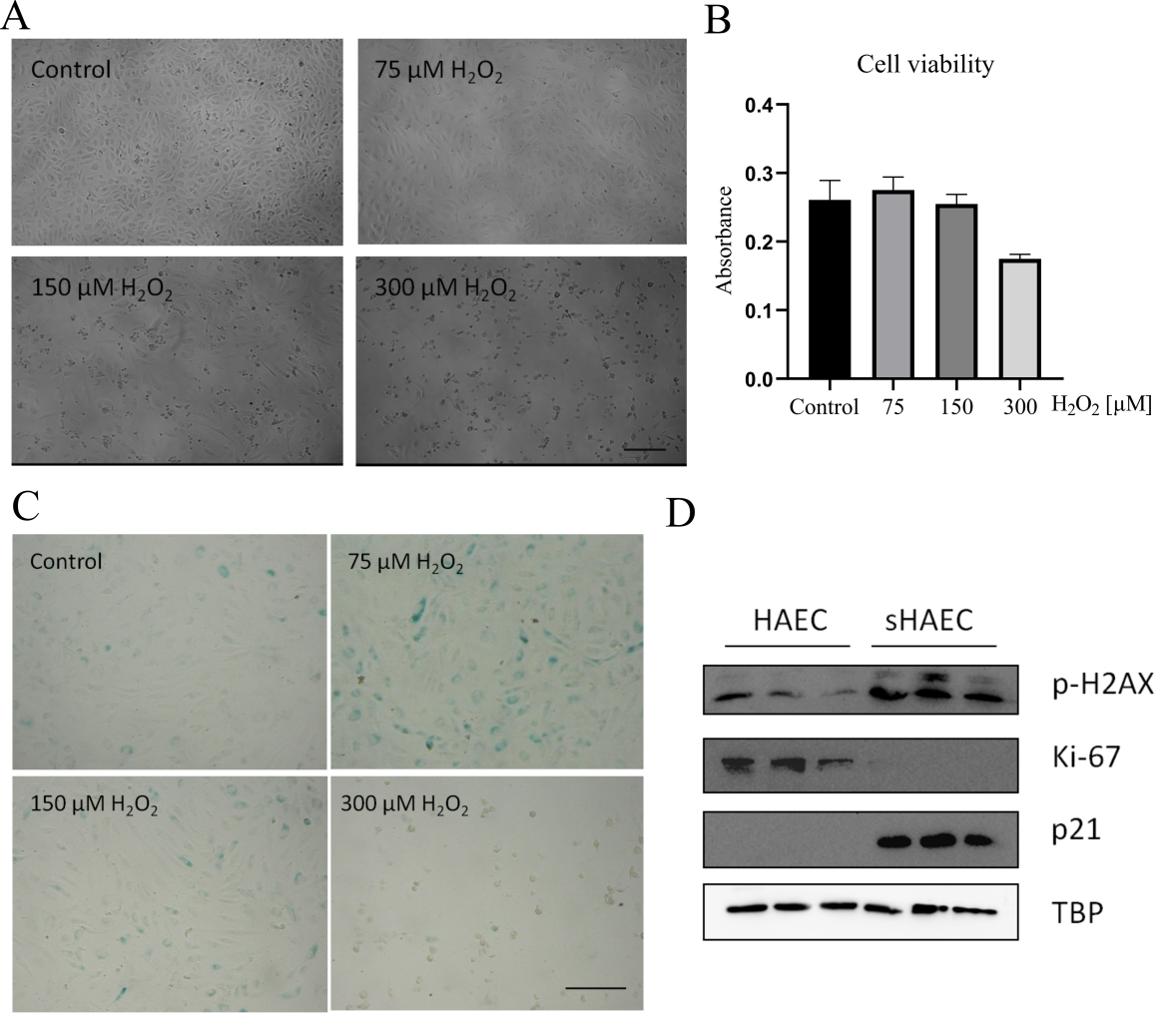


Fig. S3 Effect of different concentrations of H_2_O_2_ on HAEC viability and senescence rate. (A) HAEC morphology examined by light microscopy. (B) HAEC viability in MTT assay. (C) Representative bright field microscopy pictures of SA-β-gal staining. (D) Western blot analysis of histone phospho-H2AX (p-H2AX), CDKN1A (p21) and Ki-67 protein level in control and senescent HAEC (sHAEC). TBP was used as a loading control. Scale bar: 50 μm.


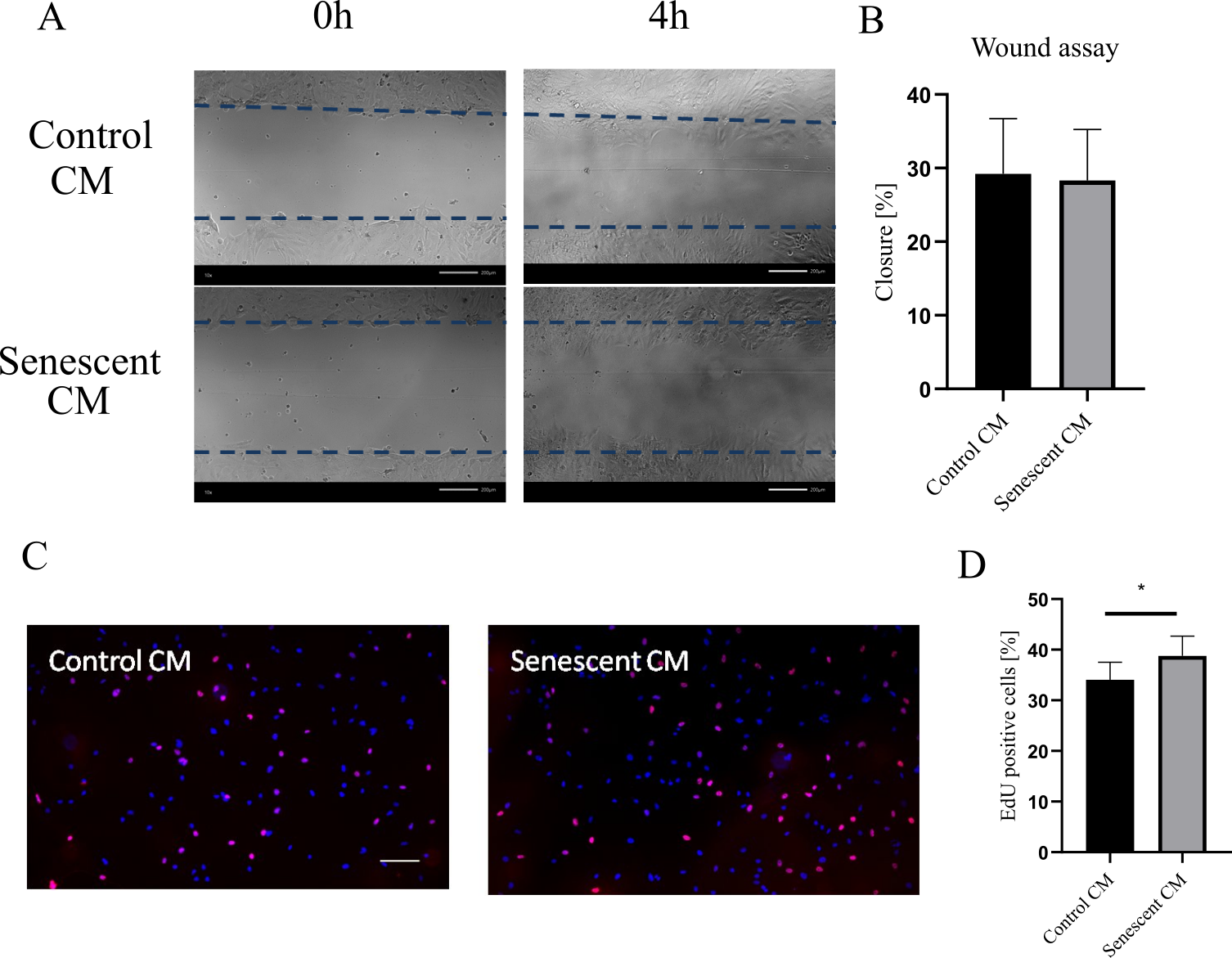


Fig. S4 Migration and proliferation of hAdv cells in response to CM from control and senescent HAEC. (A) Representative phase-contrast microscopy pictures at 0 and 4 hours after scratch generation. (B) The quantification of scratch closure after 4h. (C) Merged images showing colocalization of EdU (red) and Hoechst 33342 (blue). (D) The quantification of EdU positive nuclei as a percentage of all nuclei. Statistical analysis performed with Mann-Whitney test (*p<0.05). Scale bar: 200 μm.


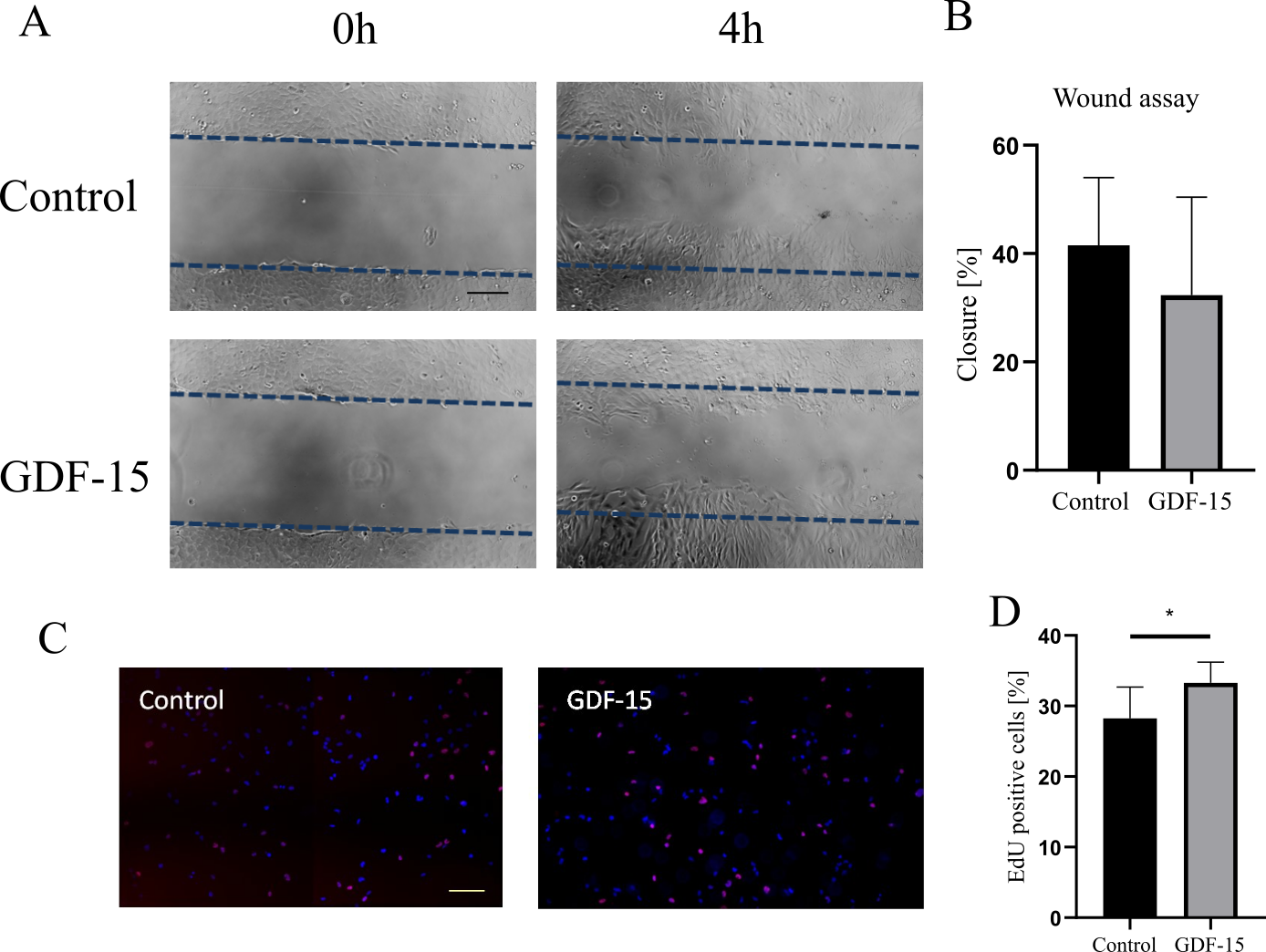


Fig. S5 Migration and proliferation of hAdv cells in response to rhGDF-15. (A) Representative phase-contrast microscopy pictures at 0 and 4 hours after scratch generation. (B) The quantification of scratch closure after 4h. (C) Merged images showing colocalization of EdU (red) and Hoechst 33342 (blue). (D) The quantification of EdU positive nuclei as a percentage of all nuclei for control and GDF-15- treated hAdv. Statistical analysis performed with Mann-Whitney test (*p<0.05). Scale bar: 200 μm.


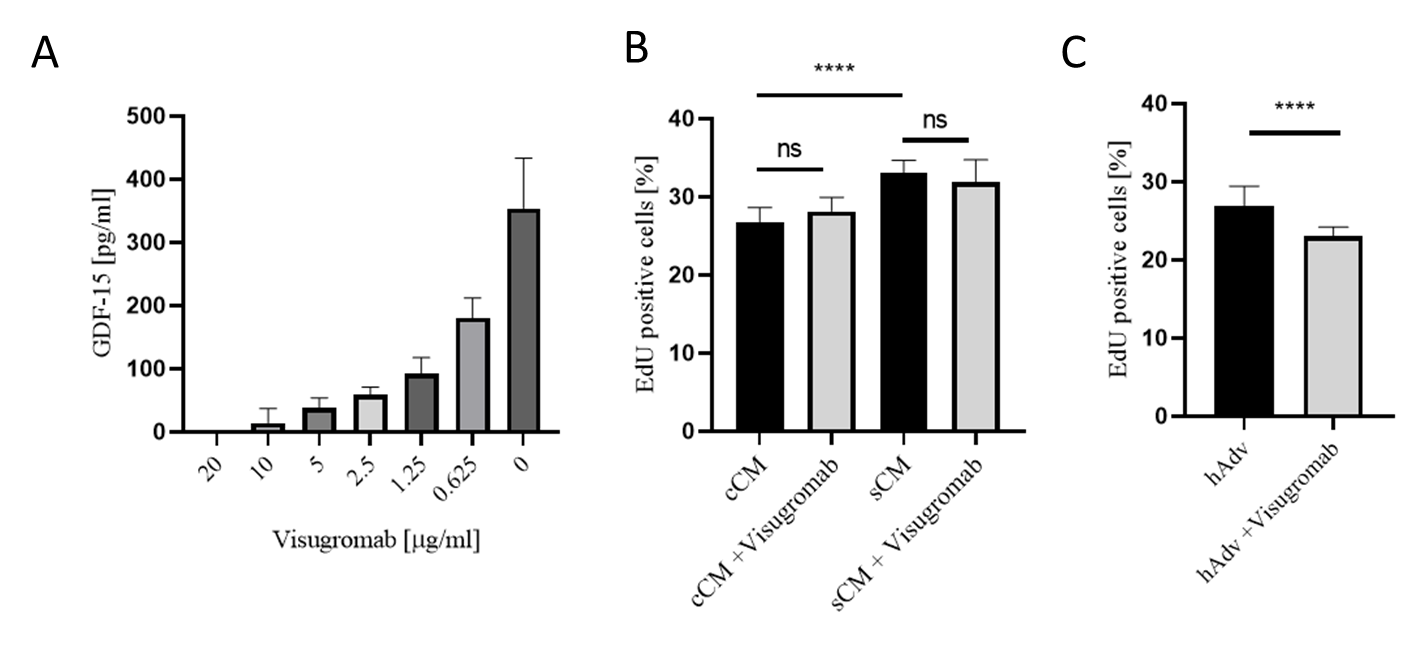


Fig. S6 Effect of GDF-15 neutralization on hAdv cells proliferation. (A) GDF-15 protein level measured by ELISA in CM following 1h incubation with different concentrations of Visugromab. (B) The quantification of EdU positive nuclei as a percentage of all nuclei for hAdv cultured in control CM (cCM) and senescent CM (sCM) with or without Visugromab (20 μg/ml). (C) The quantification of EdU positive nuclei as a percentage of all nuclei for hAdv cultured in control conditions (hAdv) or in the presence of Visugromab (20 μg/ml) in culture medium. Statistical analysis performed with Mann-Whitney test (****p<0.0001).


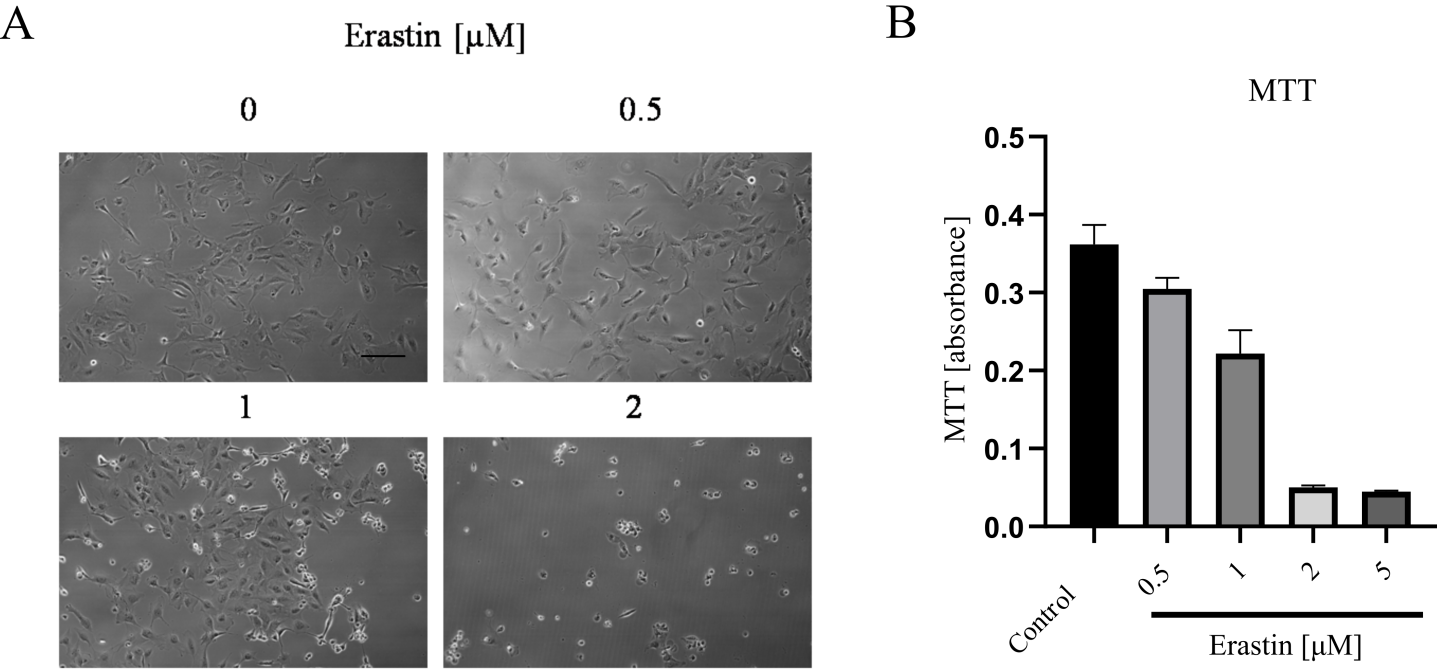


**Fig. S7** Effect of different concentration of erastin on hAdv cells viability. (A) hAdv cells treated with different concentrations of erastin for 24 h and examined by light microscopy. (B) The effects of 0-5 µM erastin on hAdv cells viability in MTT assay. Scale bar: 50 μm.


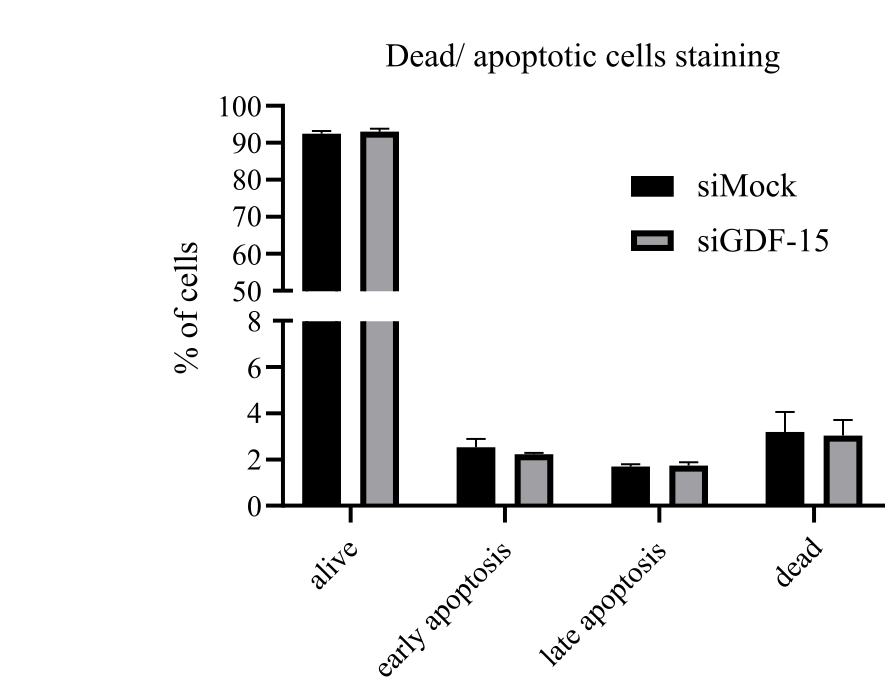


**Fig. S8** Effect of GDF-15 silencing on hAdv cells viability. The percentage of viable, apoptotic and dead hAdv cells treated with siMock or siGDF-15 based on AnnexinV and PI staining.
